# Can predicting future pregnancies after loss personalise miscarriage care?

**DOI:** 10.1016/j.lanepe.2025.101394

**Published:** 2025-07-18

**Authors:** Lara Catherine Morley, Tina Jayne Shillito

**Affiliations:** a Department of Obstetrics and Gynaecology, Leeds Teaching Hospitals NHS Trust, UK; b Leeds Institute of Cardiovascular and Metabolic Medicine, University of Leeds, UK

Recurrent miscarriage has been under-served as a research field and there are few long-term studies of future pregnancies.^1^^,^^2^ High-quality data to share with patients about their prognosis is lacking, and so the development of validated tools is welcome.

In this issue of *The Lancet Regional Health–Europe*, Constandina Koki and colleagues recruited a cohort of couples at three sites in the South of England and their future pregnancy outcome data were prospectively recorded.^3^ Over a 5-year period, 1201 couples were registered, and 942 completed the 3-year follow up. Women who experienced one or more pregnancy losses were included, which was notable as the definition of recurrent miscarriage varies across international guidelines creating heterogeneity in studies^4^^,^^5^ although the average number was 3.43 in keeping with the traditional definition of recurrent loss.

The authors group their findings into two prediction models: viable pregnancy, and time to pregnancy. In the first, they found increased maternal age, raised body mass index (BMI >25 kg/m^2^), and patients with polycystic ovary syndrome (PCOS) were more likely to experience future pregnancy loss.^3^ Interestingly, there was a progressive decrease in viable pregnancy probability from only modestly increased BMI (>25 kg/m^2^) with an odds ratio 0.97 per kg/m^2^ (CI 0.94–0.99, p = 0.02). Number of previous miscarriages was also significant, with reduction in viable pregnancy probability by 22.7% in women with ≥3 pregnancy losses. Cumulative effects are described; for example, combining advanced age and raised BMI covariates further reduced the likelihood of successful outcomes.^3^

It is well established that chromosomally abnormal conceptions increase with maternal age, whilst a higher number of previous pregnancy losses suggests alternative underlying aetiologies.^6^ Likewise, raised BMI is known to increase the chances of miscarriage in both pregnancies conceived naturally and with assisted reproduction.^7^ Hypothesised mechanisms include the impact of chronic inflammation, insulin resistance and disturbances to endometrial receptivity.^8^ Similarly, PCOS is known to increase the risk of pregnancy complications, including miscarriage, which was recently demonstrated in a large high-quality meta-analysis.^9^ Here, 44 studies of women with PCOS found higher odds of miscarriage compared to women without (OR 1.49, 95% CI 1.20–1.85), including a subset that were age- and BMI-matched.

Of those who completed the 3-year follow-up, 10.7% (101/942) did not conceive, forming a subfertility subgroup.^3^ The background risk of subfertility is 1:6 couples (∼20%^10^), and so these couples were not more likely to have difficulty conceiving. The link between recurrent miscarriage and subfertility is debated—while they may share aetiologies, including impaired endometrial receptivity, they can also be distinct. Some women with recurrent miscarriage may even be ‘superfertile’.^11^ This underscores the need for tailored care in miscarriage clinics, with timely referrals to fertility services based on individual needs.^2^

The time to pregnancy model found advanced maternal age, raised BMI, smoking, and fewer previous pregnancies delayed the likelihood of conception, which supports existing literature. The novelty of this study is using these parameters to develop predictive models for the probability that the next pregnancy is viable, and for time to next pregnancy. This enables clinicians to provide patients with a framework to guide expectations. The development of the Tommy's Miscarriage Support Tool and its accessibility on the Tommy's website (www.miscarriagetool.tommys.org ^12^) is a beneficial step towards involving patients in their care. This approach is supported by NHS England, which advocates for technological and innovative ways for patients to take an active role in their health.^4^ The availability of quantitative data may also facilitate joint decision-making about emotive issues, such as BMI, although resources to support healthy lifestyle choices remain inadequate.

While personalised care is advancing across medical specialties, progress in recurrent miscarriage has been limited by a lack of high-quality evidence. Personalisation involves identifying patient subgroups, such as those with subfertility.^2^^,^^3^ This group is highly heterogeneous, including couples with abnormal sperm tests, sexual dysfunction, and tubal disease. For in vitro fertilisation (IVF) patients, number of embryo transfer cycles and use of prenatal genetic testing of embryos for aneuploidy (PGT-A) may alter their miscarriage probability. Further research is needed to optimise care, as both recurrent miscarriage and subfertility are independently associated with adverse pregnancy outcomes.

Development of the models could occur through inclusion of participants from more diverse backgrounds as most patients were of White British ethnicity. Likewise, refinement by gestation of miscarriage and results of investigations, such as antiphospholipid antibody and thyroid testing, could help to improve model accuracy. Although this study was large, we noted from the ISRCTN registry that the target number of participants was 9000. Increasing the number and representation of respondents could increase the area under the curve for each model from 0.54 (95% CI 0.55–0.76) for pregnancy viability and 0.60 (95% CI 0.50–0.70) for time to pregnancy.

This study, and the development of the miscarriage support tool,^12^ represents a step towards personalisation in miscarriage care, and provides a framework for clinicians to guide patient expectations and plan their treatment.

## Contributors

LM: writing–original draft; TJS: writing–review & editing.

## Declaration of interests

LC Morley is a NIHR-funded Clinical Lecturer and has current research projects supported by Tommy's The Baby Charity.
